# A Case of Puerperal Eclampsia, with Remarks

**Published:** 1880-05

**Authors:** Joseph Eve Allen

**Affiliations:** Augusta, Ga.


ATLANTA
Medical and Surgical Journal.

VOL. XVIII.] MAY—1880. [No. 2.

### Original Communications.

### A CASE OF PUERPERAL ECLAMPSIA, WITH REMARKS.

BY JOSEPH EVE ALLEN, M.D., AUGUSTA, GA.

(Read before the Augusta Medical Society.)

The following clinical history of a case of puerperal eclampsia is worthy of record, for the reason that it presents symptoms which are unusual and peculiar, and illustrates fully the great value of certain remedial agents whose place in the therapeutics of this affection is at present unsettled in the opinion of the profession.

Jane S., mulatto, aged 20 years, came under my care in the latter part of August, 1879, when about seven and a half months gone in her second pregnancy. She then suffered from nausea and vomiting, which was greatly increased by the ingestion of food, a burning sensation referred to the epigastric region which, though always present, was so much increased at night that it prevented sleep, and dyspnœa, which was also worse at night, and made rest in the recumbent posture impossible. With the exception of an occasional slight rush of blood to the head, there was no cerebral disturbance. She was extremely thin and anæmic; there was, however, no edema, either of the face or extremities; the urine was secreted in small quantity, highly colored, and loaded with albumen. This patient had been affected thus for about a month previous, during which time she was subjected to treatment for dyspepsia.

I prescribed acetate potash with infusion of digitalis and sub-nitrate bismuth with saccharated pepsin. This treatment was of such apparent benefit that in a short time the case was discharged.

After the lapse of about fifteen days this patient came again under observation, now suffering from the usual cerebral disorders attendant on uræmic poisoning of the blood; the dyspnœa and gastric troubles had returned, but not so severe as before; there was great irritability of the bladder, and the urine passed was scanty, thick, dark and highly albumenous, yet there was no edema of the face or limbs perceptible. The diuretic mixture was continued and bromide potassium and hydrate chloral prescribed in addition; hip baths and sinapism to the lumbar region were also ordered; the advisability of venisection was considered, but deferred in consequence of the extremely anæmic condition of the patient.

There was no appreciable change in this patient's state until labor commenced at one o'clock in the morning of September 15th, one month before the calculated time for her confinement. On arrival I found her nervous and excited, complaining greatly of pain in the lumbar and hypogastric regions which was not intermittent, cutting or grinding, like pains usual to the first stage, but was dull, aching, constant and increasing in severity. Vaginal examination revealed the os high up and just beginning to dilate, the vertex was presenting in the first position; there was marked vesical irritation, and the urine voided was so dark and thick that it was at first mistaken by the nurse for the "show." I administered one drachm of hydrate of chloral in doses of fifteen grains, repeated every quarter of an hour. This had the effect of quieting somewhat the nervous excitement and decidedly diminishing the intensity of the pain. About six A.M., while sitting up in bed, she was seized with a violent convulsion, which was promptly controlled by the inhalation of chloroform. Dr. Jos. A. Eve was then called in consultation. On his arrival the patient was in an unconscious condition, but could be partially aroused, and when aroused was rational, and stated that she was free from pain. She remained in this condition for about an hour, during which time forty-five grains of hydrate chloral was given in doses of fifteen grains every fifteen minutes; another convulsion then came on; after this had been controlled by chloroform at Dr. Eve's suggestion, she was bled; the blood flowed freely, was thin and clotted slowly, and was stopped only with the greatest difficulty after over a pint had been taken. After the venisection the patient became perfectly conscious and remained so, sleeping occasionally from the effect of the hydrate chloral and chloroform until near ten o'clock A.M., when there was a recurrence of a slight convulsion; consciousness was soon regained, however, and at twelve o'clock, the os being completely dilated, the membranes were ruptured and in less than ten minutes a fully-developed eight months' child was born, the mother being perfectly sensible during the time of delivery. Very little blood was lost in the third stage, for the placenta being delivered by Crede's method, the womb contracted firmly. Two slight convulsions, both of which readily yielded to chloroform, occurred immediately after the birth of the child; a quarter of a grain of sulphate morphia was then given by hypodermic puncture, and an hour afterward the patient was left sleeping quietly. On my return at four P.M. she was awake and talking rationally; while talking she was taken with a violent convulsion. The convulsions then recurred at intervals of about two hours, during which time the patient was in a semi-conscious condition until half past nine o'clock P.M., when deep coma supervened. From the time of delivery until the occurrence of the last convulsion a drachm and a half of hydrate chloral, two punctures of sulphate morphia (a fourth of a grain each,) and over eight ounces of chloroform were administered. At eleven o'clock the patient was deeply comatose, the breathing was stertorous and irregular, and in rate only about eight inspirations to the minute; a puncture of the sixtieth of a grain of sulphate atropia was then given, which had the effect of rendering the respirations regular and increasing the rate to about sixteen in the minute. About an hour after the atropia had been given, and while the woman was still in this state of coma, the vein which had been opened in the morning began to bleed again and was permitted to do so until several ounces had been abstracted.

The patient remained unconscious for about twenty-four hours, but took nourishment freely. During this time ten grains of calomel, twenty grains sulphate quinia and citrate potash, in doses of thirty grains every two hours, were given. On the morning of the third day after delivery she waked up and remained sensible all day, talking almost incessantly in a quick, cross, irritable manner, but complained of no pain except that usually attendant on the establishment of lactation. The urine, which was drawn by the catheter, was clear and normal in quantity, and the bowels moved frequently from the action of the calomel. The sulphate quinia and citrate potash was continued, but no hydrate chloral or sulphate morphia was taken. At ten o'clock P.M. a teaspoonful of "Dugas' astringent mixture" was given to check the bowels; after this the patient went to sleep and slept until about one o'clock the next morning, when she awoke with a slight convulsion which was immediately followed by deep coma. When I reached her, about half an hour after the convulsive seizure, she was perfectly comatose and her breathing was irregular, stertorous and between six and eight inspirations to the minute; a puncture of the forty-eighth of a grain of sulphate atropia was given, which increased the rate of respiration to eighteen per minute; after this mustard was applied to the chest and extremities, cold water to the head and a blister to the back of the neck. In about two hours, or as soon as the blister began to draw, the patient commenced to arouse and had no return of the eclampsia. She convalesced slowly, but is now almost restored to health; there exists, however, almost complete atrophy of the muscles of the hands and forearms.

The infant died of spasms, probably caused by indigestion, on the sixth day after its birth.

The points of interest in the clinical history of this case are :—

1. *The continuance of nausea and vomiting during the latter months of pregnancy.*—In the *American Journal of Obstetrics*, Jan. 1879, Dr. Richardson, of Boston, calls attention to the fact that the continuance of nausea and vomiting during the latter months of pregnancy is often indicative of nephritic disease with consquent uræmia, and he rightly urges the necessity, in all such cases, of first examining the urine chemically before attempting to treat this symptom as dyspepsia or a return of the reflex disorders of the earlier months.

In the present state of our knowledge it is impossible to say positively whether this nausea and vomiting is due to the circulation through the brain of blood poisoned by the accumulation of excrementitious material or whether it is a morbid reflex action anteceding or becoming developed at the same time with the disturbance of the renal function, and having with it as a common cause, some hidden source of puerperal irritation. It is probable that the last is the true explanation, for if closely observed it will be found that cases of puerperal eclampsia frequently occur in which, as in the case above reported, the gastric disorder precedes for weeks the headache, blindness, dizziness and œdema symptoms characteristic of uræmic blood poisoning. The persistence of nausea and vomiting during the latter months of pregnancy is therefore a condition of great pathological significance, being highly indicative of acute nephritis and prognostic of puerperal eclampsia.

2. *The recurrence of the eclampsia on the third day after delivery.*—The recurrence of the eclampsia on the third day after delivery, at a time when the urinary organs were acting normally and the woman had been perfectly conscious for over twelve hours, was doubtless due to the systemic excitement attendant on the establishment of the lacteal secretion, and worthy of note in that the convulsive seizures may be induced in women whose nervous systems are morbidly impressible by various causes of direct or indirect irritation, or as Rosenthal states,* puerperal eclampsia is really a reflex epilepsy.

3. *The therapeutic value of hydrate chloral in the treatment of puerperal eclampsia.*—Attention is called to the benefit derived from the use of hydrate chloral in this case especially for the reason that although now the use of this drug is advised in almost every standard work on obstetrics, and the current medical literature of the day is full of reports of the good results obtained from its employment, still Dr. Fordyce Barker has recently expressed himself adversely to it,† stating that he has found that in puerperal eclampsia hydrate chloral does not, like chloroform, allay, but that he is strongly suspicious that it excites nervous irritability. This opinion coming, as it does, from one of the highest authorities in this country, and based, as it is, on such a vast amount of clinical observation and research, demands most careful consideration and is justly entitled to the highest respect, and therefore the place of hydrate chloral among our resources for the treatment of this affection is at present a matter which is only to be determined by the study and comparison of individual experiences.

Dr. Wm. Goodell, in remarks before the Obstetrical Society of Philadelphia,‡ advocates the use of hydrate chloral in puerperal eclampsia, and reports several cases in which the convulsions were absolutely controlled by the drug; and again, at the last meeting of the same society, he said‖ that he considered it the best single remedy for the treatment of this disease, and that he had never lost a case to which hydrate chloral had been administered.

Dr. Jos. A. Eve has used hydrate chloral in his practice ever since its first introduction into obstetric medicine. In the treatment of puerperal eclampsia he regards this drug as one of the most valuable of remedial agents. Its administration has, in his experience, always been attended with the most satisfactory results. In several cases

*Diseases of the Nervous System.

†Puerperal Diseases, page 120.

‡*American Journal of Obstetrics*, Vol. IX., page 495.

‖*American Journal of Obstetrics*, April, 1880.

where puerperal eclampsia was immediately threatened, Dr. Eve believes that he has prevented its occurrence by giving hydrate chloral during the first stage of labor until its hypnotic effect was produced. It has been his observation that the use of this drug during labor calms nervous excitement, lengthens the intervals, and induces rest between the pains, and thus strengthens and renders more efficient the uterine contractions, while at the same time the woman is saved much suffering and exhaustion. As an anæsthetic during delivery it will of course never take the place of sulphuric ether or chloroform, but he considers hydrate chloral peculiarly applicable at a period when those agents cannot be used to advantage, viz , during the first stage when a drug is required which is continuous in its action and does not necessitate the constant personal supervision of the medical attendant.

Dr. Richardson, of Boston, in a paper read at the first meeting of the American Gynecological Society,* has recorded his views on the use of hydrate chloral in obstetrics, and especially as to its good effect in the treatment of puerperal eclampsia.

If the systemic action of hydrate chloral be studied in connection with any one of the many theories which have been advanced to explain the etiology of puerperal eclampsia, it will be seen that, in accordance with each, its use is rationally indicated; but as it is impossible here to consider these opposite and contradictory theories held by different pathologists, the following synopsis of the views of Dr. H. F. Campbell, drawn from his paper on "Blood-letting in Puerperal Eclampsia,"† is taken as expressing concisely and completely, and interpreting most reasonably, all that is now known of the pathology of this affection—that is, that puerperal eclampsia is induced by several widely different morbid processes, all culminating in the one overruling proximate cause, the pathological condition of nervous irritation. Now, as hydrate of chloral is proved by physiological experimentation‡ and clinical

---

*Transactions American Gynecological Society—Vol. 1.

†American Journal of Obstetrics, Vol. IX.

‡See the researches of Labbee, Rayewsky, H. C. Wood and other physiologists, H. C. Wood's Therapeutics, Materia Medica and Toxicology.

observations to be, when given in full doses, a most certain depresso-motor, acting as a depressant upon the centres of the base of the brain and spinal cord, thus allaying reflex excitability and subduing nervous irritation, it is manifest that its use is indicated in puerperal eclampsia as a most certain remedial agent, secondary in importance only to the more prompt cerebro-spinal sedatives, venesection and chloroform.

4. *The great value of blood-letting in puerperal eclampsia, and especially in apparently anemic cases of the affection.*— This case illustrates the good effect of blood-letting, especially in apparently anemic cases of puerperal eclampsia, for the woman was thin, emaciated and seemingly almost exsanguined; in fact, her condition was such that Dr. Eve, who has long regarded venesection as one of the most prompt and efficient means at our command for the control of puerperal eclampsia,* hesitated before advising in her case a resort to this measure. The abstraction of blood was, however, followed by the most marked and immediate benefit, the explanation of which is found in the fact that, as Barker says,† "In hydræmia there may be an excess in the quantity of blood—a kind of serous plethora, resulting in great disturbance of the circulation and local congestions, which will be overcome by moderate venesection, followed by more nutritious animal diet and the use of iron and other tonics."

In the same article, referring to this subject, he very forcibly remarks, "It has seemed to me there is some liability to err in the neglect of blood-letting, from the feeling that this measure should never be resorted to unless the patient was in a sthenic condition. But some of the most striking instances of its usefulness have occurred under my observation where the patient was extremely anæmic."

Dr. H. F. Campbell has demonstrated the rationale of therapeutic action of vensection in puerperal eclampsia. In his article above referred to, he has proved that blood-letting, by reducing vascular pressure, relieves congestion

*Southern Medical and Surgical Journal—Vol. III.

†Blood-letting as a Therapeutic Resource in Obstetric Medicine.

of the brain and spinal column, thus acting as a cerebro-spinal sedative, while at the same time, by lessening turgescense of the kidney, it aids most materially in the elimination of the poison from the blood. Observation of cases, not only of puerperal but of non-puerperal uremic eclampsia, has led me to believe that although this sedative action on the nervous system is most strikingly apparent and of great importance in the prompt control of the convulsive seizures, still that the abstraction of blood is frequently of as much, if not of more, permanent benefit in re-establishing the renal functions.

5 *The use of sulphate atropia in puerperal eclampsia.*—The good results obtained from the hypodermic administration of sulphate atropia in this case, proves its value as a respiratory stimulant during the comatose state supervening after puerperal convulsions. It may be urged that when first employed it acted simply as a physiological antidote to the hydrate chloral and other drugs which had been taken. This I am not prepared wholly to deny; but the coma on the third night after delivery was, without doubt, due to the eclampsia, for it could not possibly have been caused by the small quantity of opium given four hours previous in the Dugas mixture; and it was in this last instance that the great benefit of sulphate atropia was most evident.

